# Intensive Lipid-Lowering Therapy Following Acute Coronary Syndrome: The Earlier the Better

**DOI:** 10.3390/jcdd12080300

**Published:** 2025-08-04

**Authors:** Akshyaya Pradhan, Prachi Sharma, Sudesh Prajapathi, Maurizio Aracri, Ferdinando Iellamo, Marco Alfonso Perrone

**Affiliations:** 1 Department of Cardiology, King George’s Medical University, Lucknow 226003, Uttar Pradesh, India; drprachi1009@gmail.com; 2 Department of Cardiology, All India Institute of Medical Sciences, Bhopal 462020, Madhya Pradesh, India; sudeshprajapathi@gmail.com; 3 Division of Cardiology and CardioLab, Department of Clinical Sciences and Translational Medicine, University of Rome Tor Vergata, 00133 Rome, Italy; maurizio.aracri@ptvonline.it (M.A.); iellamo@uniroma2.it (F.I.)

**Keywords:** LDL cholesterol, acute coronary syndromes, combination therapy, statins, PCSK9 inhibitors

## Abstract

Elevated levels of atherogenic lipoproteins are known to be associated with an increased risk of incident and recurrent cardiovascular events. Knowing that the immediate post-acute coronary syndrome (ACS) period is associated with the maximum risk of recurrent events, the gradual escalation of therapy allows the patient to remain above the targets during the most vulnerable period. In addition, the percentage of lipid-lowering levels for each class of drugs is predictable and has a ceiling. Hence, it is prudent to immediately start with a combination of lipid-lowering drugs following ACS according to the baseline lipid levels. Multiple studies with injectable lipid-lowering agents (PCSK9 inhibitors) such as EVOPACS, PACMAN MI, and HUYGENS MI have shown the feasibility of achieving LDL-C goals by day 28 and beneficial plaque modification in non-infarct-related coronary arteries. Recently, a study from India demonstrated that an upfront triple combination of oral lipid-lowering agents was able to achieve LDL-C goals in a majority of patients in the early post-ACS period. This notion is also supported by a few recent lipid-lowering guidelines advocating for an upfront dual combination of a high-intensity statin and ezetimibe following ACS. Henceforth, the goal should not only be the achievement of lipid targets but also their early achievement. However, the impact of this strategy on long-term cardiovascular outcomes is yet to be ascertained.

## 1. Introduction

Cardiovascular diseases are the leading cause of mortality across the globe [1]. There is an increasing incidence of acute coronary events and coronary artery disease in younger age groups. Along with the increasing life expectancy of the population, the overall burden of cardiovascular diseases is increasing. While primary prevention holds importance, it is equally important to prevent recurrent events in individuals with incident cardiovascular events. It is known that patients undergoing percutaneous intervention (PCI) are at an increased risk of developing recurrent events [2]. Levels of low-density lipoprotein cholesterol (LDL-C) are linearly related to the risk of cardiovascular events [3,4,5]. It is also well established that lowering LDL-C linearly lowers the risk of future cardiovascular events. Therefore, it is crucial to lower the circulating LDL-C in a timely manner and prevent increasing atherosclerosis and recurrent events. However, there are no definite guidelines on when to screen patients for LDL-C levels post-PCI and how to achieve optimal targets in this subgroup.

### 1.1. Cardiovascular Risk Post-PCI/ACS

The National Cardiovascular Data Registry of the United States, the CathPCI registry [6], recently found that one in six patients suffered a major cardiovascular adverse event within 1 year of their initial coronary percutaneous intervention. Additionally, patients with higher LDL-C post-PCI have a higher risk of cardiovascular events on follow-up. The risk of cardiovascular death, myocardial infarction, and coronary revascularization increases with increasing LDL-C levels post-PCI [7]. This followed a linear relationship between 70 and 200 mg/dL in a population-based post-PCI cohort analysis [7]. Every 10 mg/dL increase in LDL-C was associated with an almost 1.6% higher incidence of MACE at 3 years post-PCI. The Reduction of Atherothrombosis for Continued Health (REACH) registry [8] has shown that the risk of recurrent ischemic events is 4.7% in the first year after an acute event, and the residual risk increases with time. The use of statins decreases the risk of recurrent events, with the benefit proportionately greater with high-intensity statins and more intensive lipid control [9,10].

Patients with an acute coronary event are at an elevated risk of recurrent events mainly due to residual lesions in the non-infarct-related artery. If these lesions are obstructive, they undergo intervention with the aim of complete revascularization. However, non-obstructive lesions pose difficulty as they progress and may harbor a vulnerable plaque with a significant lipid burden. The PACMAN-AMI study assessed non-infarct-related arteries with multimodality intracoronary imaging in patients undergoing culprit vessel PCI for acute MI to determine the effects of early administration of Proprotein Convertase Subtilisin/Kexin type 9 (PCSK9) inhibitors [11]. Treatment with alirocumab, as compared to a placebo, in addition to statin therapy, resulted in a significantly greater regression of atherosclerosis after 52 weeks. There was a significant reduction in atheroma volume as assessed with intra-vascular ultrasound (IVUS) and the near-infrared spectroscopy (NIRS)-measured maximum lipid core burden index within a 4 mm segment. There was a significant increase in the fibrous cap thickness (FCT), as assessed through optical coherence tomography (OCT). Similarly, the HUYGENS (High-Resolution Assessment of Coronary Plaques in a Global Evolocumab Randomized Study) evaluated the effects of monthly treatment with evolocumab vs. a placebo for 52 weeks in patients with non-ST-segment elevation MI (NSTEMI). Evolocumab resulted in a greater increase in FCT and a decrease in the maximum lipid arc and macrophage index. This implies that early and intensive lipid reduction and plaque modification may result in the prevention of recurrent events [12].

### 1.2. LDL Targets

Conventional guidelines for the management of dyslipidemia recommend LDL-C levels ≤ 70 mg/dL for patients with established atherosclerotic cardiovascular disease. With the aim of continuing these benefits while further lowering LDL-C, the recent 2019 European Society of Cardiology (ESC)/European Atherosclerosis Society (EAS) Guidelines on the management of dyslipidemia recommended lowering the LDL-C to <55 mg/dL in very-high-risk patients with documented atherosclerotic cardiovascular disease (ASCVD) for both primary and secondary prevention (Class I recommendation) and to <40 mg/dL in patients with a second vascular event within 2 years (Class IIb recommendation) [13]. A 50% reduction is desired if the baseline LDL-C level is 70–135 mg/dL. The 2018 American Heart Association (AHA)/American College of Cardiology (ACC) guidelines recommend at least a 50% reduction in LDL-C and a 70 mg/dL LDL-C target in very-high-risk ASCVD patients [14]. However, the Indian dyslipidemia guidelines are a bit more aggressive and recommend a routine LDL-C goal of <50 mg/dL in ACS and <30 mg/dL in selected cases [15].

### 1.3. Current Status of Lipid (LDL-C)-Lowering

Several studies have reflected upon the suboptimal control of LDL-C in clinical practice. While some patients fail to achieve optimal LDL-C targets despite statin treatment, others are not even prescribed statins [16,17]. The failure to achieve LDL-C targets post-ACS is attributed to clinical inertia, where providers underutilize high-intensity statins and combination therapies (e.g., ezetimibe, PCSK9 inhibitors). Access limitations to advanced therapies, particularly PCSK9 inhibitors due to cost constraints, further hinder goal attainment. Patient-related factors like non-adherence (24% discontinue statins by 6 months) and perceived side effects (contributing to 37–60% discontinuations) exacerbate the issue [18]. Systemic gaps, including delayed follow-up and insufficient lipid monitoring, also play a role, with observational data showing 45% of patients remain above LDL-C targets at 4–6 weeks. Addressing these requires protocol-driven early intensification, patient education, and improving access to affordable combination therapies [19]. In a recent study, it was emphasized that only about one half of the patients had their cholesterol levels checked before 6 months post-PCI [7]. Among those who had it checked, almost half had LDL-C levels above 70 mg/dL. This reflects the lack of reinforcement among medical practitioners to reach LDL-C goals post-PCI. Among those who are prescribed statins, there is underutilization of high-dose statins [20]. This is especially common in older patients. Older patients (≥75 years) derive comparable relative risk reductions from lipid-lowering therapy (LLT) to younger patients, with a 26% reduction in major vascular events per 1 mmol/L LDL-C reduction. Notably, absolute risk reductions may be higher in older adults due to their elevated baseline risk [21]. Younger patients in primary prevention are often undertreated despite high modifiable risk factors (e.g., 77.3% smoking, 50.3% dyslipidemia in those ≤ 45 years). The 2021 ESC guidelines for cardiovascular disease prevention in clinical practice recommend statins for individuals < 50 years with SCORE2 ≥ 7.5% (very high risk) or ≥2.5% (high risk) [22]. All post-ACS patients, regardless of age, require aggressive LLT. The ESC 2021 guidelines also advocate LDL-C targets < 1.4 mmol/L (55 mg/dL) for post-ACS patients. The guidelines prioritize the SCORE2 model for risk stratification, expanding statin eligibility in younger adults. According to a recent study, among patients older than 65 years, only 48% were on high-intensity statins, while 22% were not receiving any statin therapy at all [7]. This is contrary to guidelines, which recommend high-dose statins for all age groups of patients with established CAD. Among patients with elevated levels of LDL-C on follow-up, the proportion of patients receiving high-intensity statin therapy is proportionately lower. However, despite high-intensity statins, 25% of patients may still not achieve LDL-C < 70 mg/dL [7]. Therefore, routine testing is needed on follow-up in order to escalate lipid-lowering therapy. Delays in testing or a lack of testing expose the patients to elevated atherogenic lipoproteins for a longer duration.

Several studies have highlighted the poor LDL-C target attainment in clinical practice [23,24,25,26]. In a cross-sectional survey of data collected from electronic medical records, administrative claims, and national survey databases of high-risk CAD patients from the United States, less than a quarter of patients had LDL-C levels ≤ 70 mg/dL on statin therapy [23]. Recent data from the **DA VINCI** study clearly showed that only 18% of patients achieved an LDL-C level of 55 mg/dL [24].

In a very large cohort of 57,885 statin-treated CAD subjects, the **DYSIS** study found that the LDL-C target was attained by only 21.7% [25]. Among patients hospitalized for a first or recurring coronary event, the **EUROASPIRE IV** study found only 19.3% had an LDL-C level below 70 mg/dL despite the use of statins [26]. The DYSIS II study included patients with stable coronary heart disease (CHD) or an acute coronary syndrome (ACS) from 18 countries, and surprisingly, only 29.4% and 18.9%, respectively, displayed an LDL-C level below 70 mg/dL [27]. The mean daily dose of statin was merely 25 ± 18 mg atorvastatin equivalent. Overall, although statin therapy was prescribed in more than 90% of the patients, less than one third achieved an LDL-C target of <70 mg/dL. This leads to a false sense of confidence among both the health care provider and the patient, while the benefit of adequate lipid-lowering is not achieved. It is therefore important to have guidelines to check the lipid profile of patients regularly with established CAD post-PCI.

While LDL-C remains the primary target, elevated triglycerides (TG > 200 mg/dL) confer a residual risk, particularly in patients with metabolic syndrome. The REDUCE IT trial showed that administration of icosapent ethyl 4 g daily reduced the primary composite cardiovascular endpoint by 25% (HR 0.75, 95% CI 0.68–0.83, *p* < 0.001) in patients on statin therapy with persistent high TG. There was also a significant reduction in cardiovascular death (HR 0.80, 95% CI 0.66–0.98) [28]. However, the STRENGTH trial found no benefit with omega-3 carboxylic acids, underscoring formulation-specific effects [29]. Current guidelines prioritize LDL-C-lowering first, followed by TG reduction if levels persist >500 mg/dL for alleviating the risk of pancreatitis [13,14].

The various treatment options in vogue for achieving the guideline recommended lipid targets after ACS are summarized in Table 1.

### 1.4. Benefits Beyond Statins Monotherapy

**Figure 1:**
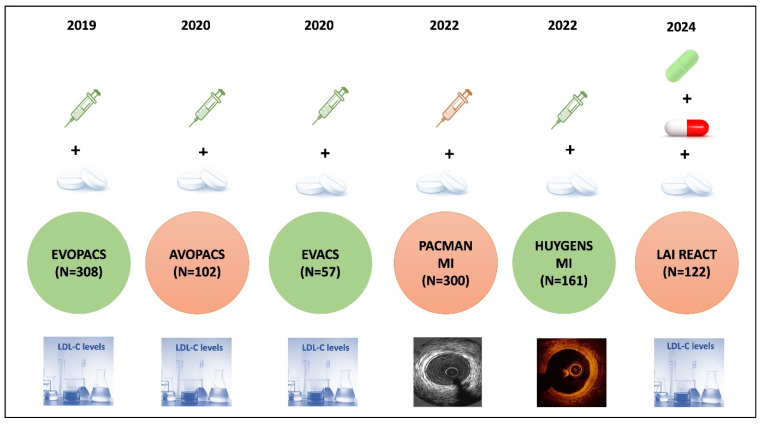
Landmark trials for early and intensive LDL-C lowering after an acute coronary syndrome. The green injections represent evolocumab, the orange injections represent alirocumab, the blue tablets represent statins, the red and white capsule ezetimibe, and the green tablet bempedoic acid. All studies have utilized PCSK9 inhibitors with the exception of the LAI REACT study, which used oral lipid-lowering therapy. In most of the studies, attainment of the LDL-C goal was the primary end point, while intracoronary imaging was utilized in the PACMAN-MI -IVUS and HUYGENS MI -OCT, respectively.

An elevated LDL-C despite high-dose statin therapy confers an elevated risk of cardiovascular events. The addition of non-statin lipid-lowering therapy (LLT) brings the highest benefit in risk reduction in this cohort. This additional LLT has extensively been studied in the form of ezetimibe and PCSK9 inhibitors (Figure 1). Randomized controlled trials (RCT) have shown that lowering LDL-C levels beyond maximally tolerated statin therapy is associated with a significant reduction in major adverse cardiovascular events, including a composite of cardiovascular death, myocardial infarction, stroke, coronary revascularization, and unstable angina [30,31,32]. The greatest risk reduction with treatment intensification is seen in those with LDL-C > 100 mg/dL [10]. Although international guidelines recommend testing for lipids at 4–6 weeks after an acute coronary event, it is worth realizing that an elevated LDL-C is associated with enhanced risk of cardiovascular events irrespective of the indication for PCI [13,14]. So, even patients with stable CAD require aggressive lipid management following PCI. Thus, there is an unmet need for updating the contemporary guidelines for patients undergoing PCI regarding early and intensive reduction in LDL-C.

A large meta-analysis of statin trials has shown that statins decrease the risk of major vascular events by 21% per 1 mmol/L (39 mg/dL) reduction in LDL cholesterol [5]. The benefits are seen as early as the first year of therapy. To decrease the exposure to elevated levels of atherogenic lipoproteins, it is prudent to start intensive therapy with multiple lipid-lowering agents early after PCI. When added to statin therapy, ezetimibe reduces LDL-C levels by an additional 24%, hence causing a reduction in cardiovascular risk [30,33]. Recently, PCSK9 inhibitors have been evaluated for lipid-lowering in the early phase (within 24 h) following PCI [34,35]. In the EVOPACS study, treatment with evolocumab in addition to statin therapy led to a reduction in LDL-C levels to <70 mg/dL in almost 95% patients and even to <55 mg/dL in >90% of the patients as early as 8 weeks [34]. While with statins alone, approximately only one-third of the subjects could achieve an LDL-C level of <70 mg/dL this early. In a Japanese population with ACS, the addition of evolocumab to pitavastatin therapy led to LDL-C levels < 70 mg/dL and <55 mg/dL in >95% cases at 4 weeks.

In the EVACS study, a single dose evolocumab initiation on the top of high-intensity statins in the hospital early after ACS has been shown to significantly reduce LDL-C as early as at hospital discharge [35]. At discharge itself, >80% patients were able to achieve the ACC/AHA guideline mandated LDL-C target level of <70 mg/dl. Apart from LDL-C, non-HDL-C and ApoB levels were also significantly lower at discharge. 

However, there is definite inertia for the use of adjuvant lipid-lowering agents beyond statin therapy. In the DYSIS II, 82.3% subjects were on statin monotherapy. Ezetimibe in combination with a statin was used in 10.5% of patients, while another 5.8% were on combination of a statin and other non-statin drug [25].

Statins, beyond their well-established lipid-lowering properties, exhibit a range of pleiotropic effects. These effects include anti-inflammatory actions, improvement in endothelial function, plaque stabilization, reduction in oxidative stress, and modulation of immune responses, all of which may contribute to improved post-operative outcomes in valve surgery patients [36,37]. The CANTOS trial confirmed that targeting inflammation (via IL-1β inhibition) reduces MACE independently of LDL-C, supporting dual-pathway management [38]. The post-ACS follow-up should include a lipid profile at 4–6 weeks and then every 3–6 months until the target LDL-C is achieved [13,14].

Lifestyle interventions, including Mediterranean-style diets (rich in vegetables, nuts, and whole grains), aerobic exercise (3–4 sessions/week), and smoking cessation, achieve modest LDL-C reductions (~5–15%) but are insufficient alone for high-risk post-ACS patients [39]. Current guidelines emphasize combining lifestyle changes with early intensive statin-based therapy, as dietary and exercise modifications alone rarely meet the stringent LDL-C goals required for plaque stabilization.

### 1.5. Lipid-Lowering in a Special Scenario—Kidney Disease

Patients with chronic kidney disease (CKD)—especially after an acute coronary syndrome (ACS)—face very high cardiovascular risk. Multiple trials have shown that aggressive LDL-lowering reduces CV events in patients with CKD. In the SHARP trial, simvastatin (20 mg) plus ezetimibe (10 mg) therapy lowered LDL by 0.85 mmol/L and reduced major atherosclerotic events by 17% (RR 0.83, 95% CI 0.74–0.94, *p* = 0.002) in 9270 patients with CKD (stages 3–5, ~30% on dialysis) [40]. Importantly, a subgroup analysis found a similar relative benefit in dialysis versus non-dialysis CKD patients. However, the trial was not adequately powered to draw firm conclusions in the dialysis subset. A pooled analysis by the Cholesterol Treatment Trialists showed that the relative risk reduction per mmol/L LDL drop decreases as GFR falls, with little incremental benefit seen in end-stage (dialysis) CKD; nevertheless, the absolute risk is so high that even a modest relative effect yields a clinically important absolute benefit [41]. Accordingly, the contemporary guidelines treat non-dialysis CKD as a high or very-high risk condition. The 2019 ESC/EAS recommendations give a Class I indication to treat all stage 3–5 CKD patients (not on dialysis) with a statin (or statin/ezetimibe) [13].

By contrast, initiation of statin therapy in end-stage renal disease patients on dialysis therapy remains controversial. Large RCTs in end-stage renal disease showed no significant reduction in the composite of CV death, MI, or stroke. In the 4D trial (diabetics on hemodialysis), treatment with high-dose atorvastatin did not significantly change the primary endpoint (37% vs. 38% event rate; relative risk 0.92, 95% CI 0.77–1.10, *p* = 0.37) (except for a nominal reduction in total cardiac events, RR 0.82, *p* = 0.03) [42]. Similarly, AURORA (hemodialysis patients) found no CV benefit with rosuvastatin therapy, with 9.2 events vs. 9.5 events/100 patient years in rosuvastatin vs. placebo (HR 0.96, 95% CI 0.84–1.11; *p* = 0.59) [43]. These neutral results led the KDIGO and ESC guidelines to advise against initiating statins in dialysis patients without known ASCVD.

However, this may not be the case in the context of ACS. Observational studies suggest post-ACS dialysis patients do benefit from statins. In a Taiwanese cohort (n ≈ 3900), dialysis patients started on moderate high-intensity statins after MI had significantly lower mortality (1-year mortality-23% with statin vs. 31% without; HR-0.70 and 4-year mortality 48% vs. 55%; HR ≈ 0.76). No increase in adverse events was seen [44]. Thus, in practice, many experts recommend continuing or even initiating lipid therapy after ACS in CKD, even on dialysis, balancing the potential benefit against unclear RCT evidence.

### 1.6. Lipid-Lowering in Primary vs. Secondary Prevention

Primary prevention (no known ASCVD) relies on global CV risk assessment. CKD (even without prior MI) often categorizes a patient as high/very-high risk. For example, ESC/EAS guidelines classify all stage 3–5 CKD patients as high/very-high risk; statin (or statin/ezetimibe) therapy is a Class I recommendation in non-dialysis CKD [13]. KDIGO likewise recommends moderate-dose statins in CKD ≥ 50 years old (not on dialysis) based on risk, irrespective of LDL [45]. In general, LDL targets for primary prevention are less stringent than in secondary prevention—typically <1.8–2.6 mmol/L (70–100 mg/dL), depending on risk. The intensity of statin therapy is governed by baseline risk: diabetics or severe CKD receive high-intensity, lower-risk patients may start moderate-intensity. Newer guidelines have pushed targets lower (e.g., <1.4 mmol/L for “very-high-risk” primary patients), but by definition, primary prevention goals are tailored to risk levels and often involve risk calculators (SCORE, PCE, etc.).

By contrast, secondary prevention (after ACS or other ASCVD) mandates maximal LDL-C-level reduction. Consensus documents uniformly endorse high-intensity statins for all post-ACS patients. The ESC/EAS explicitly recommends ≥50% LDL reduction and an LDL-C goal of < 1.4 mmol/L (55 mg/dL) in very-high-risk secondary prevention, while the 2021 ACC/AHA guidelines similarly advise high-intensity statins with the addition of non-statins as needed to reach an LDL-C goal of <70 mg/dL (1.8 mmol/L) or lower [13,14]. In practice, this means the need for combination therapy is not uncommon: e.g., many secondary patients require statin + ezetimibe, and if the LDL still above the goal, a PCSK9 inhibitor is warranted. The emphasis is on “treat to target” in secondary prevention, as the outcome stakes are higher.

### 1.7. Early and Intensive Lipid-Lowering

**Figure 2:**
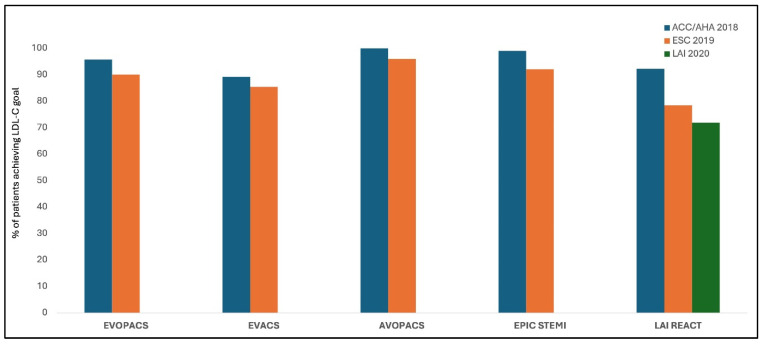
Percentage of patients achieving LDL-C goals recommended by various guidelines, after early initiation of intensive lipid-lowering therapy. [ACC/AHA—American College of Cardiology/American Heart Association; ESC—European Society of Cardiology; LAI—Lipid Association of India].

Early initiation of intensive lipid-lowering therapy (LLT) post-acute coronary syndrome (ACS) is critical, as the highest risk of recurrent events occurs within the first 30 days. The EVOPACS and EPIC-STEMI trials demonstrated that early use of a PCSK9 inhibitor (within 24–48 h post-ACS) achieved LDL-C targets (<70 mg/dL) in >90% of patients by 4–8 weeks, compared to <35% with statin monotherapy (Figure 2; [34,46]). In contrast, delayed initiation (e.g., starting at 4–6 weeks) prolongs exposure to atherogenic lipids during this vulnerable period (Table 2). The LAI REACT study showed that upfront triple oral therapy (rosuvastatin + ezetimibe + bempedoic acid; REB therapy) led to rapid and sustained LDL-C reduction: 59.3%, 62.3%, 61.6%, and 59.7% at weeks 1, 2, 4, and 6, respectively (*p* < 0.001) [47]. The target LDL-C < 50 mg/dL was achieved in 61.3% in the first week, 72.9% in the second week, 69.2% in the third week, and 65.1% in the fourth week. Thus, triple REB therapy quickly and effectively lowers LDL-C after ACS, with significant reductions seen in one week and maintained for six weeks [47,48,49].

The PACMAN MI and HUYGENS studies also demonstrated early/intensive lipid-lowering therapy leads to favorable plaque morphology, as seen on intracoronary imaging [11,12]. In the PACMAN MI trial, alirocumab use over and above high-intensity statins led to a significant reduction in the percent atheroma volume and the lipid core burden index and an improvement in the fibrous cap thickness on intracoronary imaging at 52 weeks in patients with ACS. In the HUYGENS study, evolocumab use in NSTEMI over and above statin therapy demonstrated a significant improvement in the fibrous cap thickness and maximal lipid arc in optical coherence tomography (OCT) at 52 weeks. 

The SWEDEHEART study investigated whether the timing of the combination of oral lipid-lowering therapy (statins plus ezetimibe) impacts cardiovascular outcomes in patients following myocardial infarction (MI) [50]. Utilizing data from the Swedish SWEDEHEART registry (2015–2022), the researchers compared outcomes among patients receiving ezetimibe early (within 12 weeks of discharge), late (13 weeks to 16 months), or not at all, after being discharged on statins. The study found that early combination therapy significantly improved LDL-C target attainment, with approximately 55% of patients in the early ezetimibe group reaching the target of <1.4 mmol/L (<55 mg/dL) at 1 year, despite having the highest baseline LDL-C levels. Crucially, early combination therapy was associated with a lower risk of major adverse cardiovascular events (MACE—including death, MI, and stroke) and cardiovascular death compared to delayed or no ezetimibe. For instance, at 3 years, the adjusted HR for MACE was 1.14 for late ezetimibe and 1.29 for no ezetimibe, vis-à-vis early combination therapy group. Thus, delaying or omitting combination lipid-lowering therapy results in avoidable harm, advocating for early implementation of statins with ezetimibe as the standard of care post-MI. 

A meta-analysis of nine trials (n = 38,640) found that early intensive LLT in patients with ACS significantly reduced the three point MACE risk by 12% (HR 0.88, 95% CI-0.83–0.94) and recurrent ACS by 18% (HR-0.82; 95% CI_ 0.71–0.96) compared to gradual titration [51].

Aggressive LDL-C reduction after ACS leads to sustained plaque stabilization and reduced recurrent cardiovascular events even in the long term. In the ODYSSEY OUTCOME trial, patients achieving LDL-C levels < 15 mg/dL (0.39 mmol/L) with alirocumab therapy had a 28% lower risk of major adverse cardiovascular events (MACE) over 2.8 years of follow-up compared to the placebo, even after transitioning to statin monotherapy [31]. Similarly, the FOURIER trial demonstrated that evolocumab therapy drove LDL-C levels < 20 mg/dL (0.5 mmol/L), which reduced ischemic events by 19% over 2.2 years without any increase in adverse events [32]. An open label extension of the FOURIER study with an exposure of 8.4 years revealed a 15% lower risk of a composite end point of cardiovascular death, myocardial infarction, stroke, or hospitalization for unstable angina or coronary revascularization similar to the original study [52]. However, additionally the risk of a composite of cardiovascular death, MI, or stroke was significantly reduced by 20%, as was the 23% attenuated risk of cardiovascular death.

There are no guidelines for post-PCI patients regarding monitoring and up-titration of LLT. Even in post-ACS patients, due to poor up-titration of therapy, patients experience a prolonged hyperlipidemic state. Post-ACS, the guidelines recommend lipid profile evaluation after 4–6 weeks of discharge. However, it has been found that in routine practice almost half of the patients have elevated LDL-C levels of > 70 mg/dL [7]. Unfortunately, during this early post-discharge period patients remain most susceptible to recurrent CV events. Within the first 4–6 weeks after acute coronary syndrome (ACS), the contemporary real-world data report an annualized event rate of approximately 40.9% immediately after discharge, which is nearly six times higher than the risk seen after the first year post-ACS [53]. Specifically, in the **PACSI** study, about 51.7% of patients achieved the LDL-C target of <70 mg/dL at 6–10 weeks; thus, nearly half of them remained above this threshold during a period of heightened vulnerability to recurrent events. This underscores the critical importance of early and intensive lipid-lowering therapy to reduce the risk of recurrent major adverse cardiovascular events in this high-risk window [54].

Additionally, as we now have ample knowledge regarding the efficacy of currently available LLT, the lipid reduction achievable is also predictable. So, if LDL-C is extremely high at baseline, it is unlikely that the targets would be achieved with statin monotherapy alone. Therefore, for patients with elevated LDL-C despite being on statins or even treatment naïve patients with very high LDL-C levels, it is perhaps prudent to start with combination LLT and avoid delays in attainment of the LDL-C targets. This way, we can ensure that the patients do not remain in a hyperlipidemic state in the crucial post-PCI/post-ACS period. A similar approach is already being advocated for hypertension management, where combination therapy is often started up front for patients with very high blood pressure [55].

**Figure 3:**
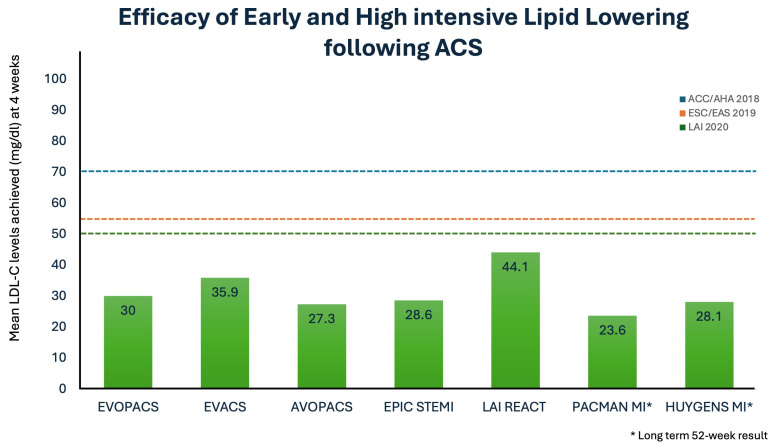
Efficacy of early and intensive lipid-lowering following acute coronary syndrome. The numerical value in each bar represents the mean LDL-C level attained on treatment in the corresponding study. The horizontal dashed lines represent the LDL-C target prescribed by contemporary lipid guidelines. With the exception of the LAI-REACT study, all studies utilized PCSK9 inhibitor therapy, while the LAI REACT study used exclusively oral LLT. [ACC/AHA—American College of Cardiology/American Heart Association; ESC/EAS—European Society of Cardiology/European association of atherosclerosis; LAI—Lipid Association of India].

We can similarly define LDL-C levels beyond which we do not expect statins alone to achieve the desired targets and, hence, start with dual or even triple LLT [56,57]. The goal of <55 mg/dL can be achieved in >90% patients with a combination of statin and evolocumab and in merely 10% with statins alone [34] (Figure 2 and Figure 3). The LAI REACT study, discussed above, evaluated the effects of concurrent triple therapy with rosuvastatin 40 mg, ezetimibe 10 mg, and vempedoic acid 180 mg, started early during hospital admission. An LDL-C level < 70 mg/dL was achieved in more than 90% patients, with almost 60% achieving an LDL-C level < 50 mg/dL as early as one week, and the effect was sustained at 6 weeks without any significant side effect [47]. Another trial studied the effect of the addition of a PCSK9 inhibitor to statin therapy in patients undergoing primary PCI for ST-elevation myocardial infarction (STEMI) [35]. There was a significantly greater reduction in LDL-C levels at 6 weeks with >90% patients achieving the LDL-C < 55 mg/dL with three doses of alirocumab, as compared to 56.7% with high-intensity statins alone. This highlights the substantial residual lipid burden with a single lipid-lowering agent in the immediate period following an episode of ACS.

We need to ensure the measurement of plasma lipids on hospital admission for an ACS/PCI, as well as 4–6 weeks later or even earlier. It is apparent that not all institutions have plasma lipids as a routine test for all such hospitalizations. Additionally, less than half have it checked even at 6 months post-PCI [7]. Even when checked, it is difficult to convince the patients of their individual goals for LDL-C, rather than simply as numbers defined by the laboratory reference values. Most of the physicians are also hesitant in up-titrating the lipid-lowering therapy and explaining to the patients about their individualized goals. In a study, it was found that of those patients who were eligible for LLT but not on statin therapy, 60% were not prescribed drugs by the physician, and 10% of patients themselves rejected it [58]. Often physicians, as well as patients, are over-apprehensive about the side effects of high-dose statins and are reluctant to prescribe the intake of such high doses. Many times, there is early down-titration of statin therapy, with slight muscle aches only [59]. It is prudent to start alternative LLT or combination therapy with low doses of statins, if there is evidence of true statin intolerance. Instead, what is more commonly practiced is the cessation of statins and then slow attempts at initiation with their low doses. This deprives the patients of the benefits of lipid-lowering, which could be achieved with an alternative agent.

If the LDL-C levels are <120 mg/dL in a statin naïve patient undergoing PCI, high-intensity statin may be started with an aim to achieve a 50% LDL-C reduction to a target <55 mg/dL. However, if the LDL-C in such a patient is 120–300 mg/dL, it is apparent that the target is less likely to be reached with statins alone [60], which would lead to prolonged exposure to elevated LDL-C levels. So, instead of the incremental approach of adding drugs on follow-up, high-intensity statin therapy can be combined with ezetimibe during hospital admission to obtain a 60–70% reduction in LDL-C levels (target < 55 mg/dL). In any patient with extremely high LDL-C levels > 300 mg/dL, triple therapy (statin + ezetimibe + bempedoic acid/PCSK9 inhibitor) might be started in hospital to obtain a >80% reduction in LDL-C in order to reach the target of <55 mg/dL. In a patient already on statins, with LDL-C levels above the desired target of 55 mg/dL but <100 mg/dL, it is advisable to intensify to the maximally tolerated statin. However, in such a patient, if the LDL-C levels are 100–300 mg/dL despite being on a statin, a combination therapy of a high-dose statin and ezetimibe should be initiated in hospital (Figure 3). This approach might help in decreasing the atheroma volume and plaque regression, along with stabilizing the plaques and, hence, reducing the event rates post-PCI [61].

In line with the concurrent evidence discussed, the Lipid Association of India (LAI) 2023 Consensus statement IV on cardiovascular risk assessment and lipid management advocates the upfront use of high-intensity statin therapy and ezetimibe in ACS patients irrespective of the baseline LDL-C levels [62]. The guidelines also demonstrate an aggressive stance by recommending a repeat lipid testing at 2 weeks for the intensification of lipid-lowering therapy, as discussed above. A similar approach is taken in a joint position statement from the Acute Cardio Vascular Care (ACVC) association, European Association of Preventive Cardiology (EAPC), and the European Society of Cardiology (ESC) Working Group on Cardiovascular Pharmacotherapy [39]. They also advocate a strike early and strike hard approach and recommend initiation of LLT in patients with ACS with a dual combination of high-dose statin and ezetimibe. However, the position statement is bit less aggressive compared with the LAI consensus statement IV in adopting the conventional waiting period of 4–6 week for a repeat lipid profile after initiation of dual-combination LLT. 

## 2. Conclusions

**Figure 4:**
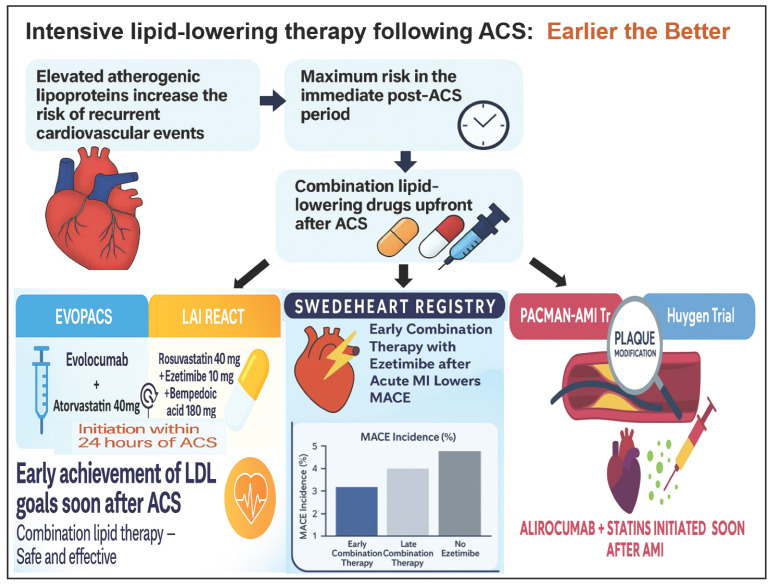
Central illustration representing the benefits of early aggressive lipid-lowering early after ACS.

With an aim to minimize the risk of future cardiovascular events in patients with established ASCVD, it is crucial to significantly and rapidly lower LDL-C levels, thereby limiting their exposure to atherogenic lipoproteins (Figure 4). Elevated LDL-C is linearly related to the event risk, and prolonged exposure worsens this risk. Despite established guidelines recommending intensive lipid-lowering, many patients do not achieve optimal LDL-C targets, with studies showing low rates of guideline adherence and target attainment. This is partly due to delays or lack of lipid testing post-PCI and reluctance to intensify therapy.

The following steps are recommended for improvement in the near future:

- Guidelines should mandate baseline lipid testing during admission for ACS/PCI.
- Early initiation of intensive lipid-lowering therapy is necessary. This may involve starting combination therapy (dual or even triple) up front after ACS/PCI, depending on the baseline LDL-C levels, rather than relying on a gradual escalation approach. This approach is supported by recent consensus statements.
- Individualized lipid targets should be explained to every patient, and closer follow-up is needed to ensure targets are met and allow for timely therapy intensification.
- Efforts should be made to overcome clinical inertia regarding the use of adjuvant lipid-lowering agents beyond statins and address concerns about high-dose statin side effects that lead to under-treatment or early discontinuation.

Implementing these measures can help ensure earlier achievement of LDL-C targets in a greater proportion of patients and potentially reduce the future CV event rates.

## Figures and Tables

**Table 1:** Treatment options for post-ACS lipid management.

Therapy	Mechanism	Advantages	Limitations	Key Trials
High-intensity statin	HMG-CoA inhibition	Low cost, mortality benefit	Myopathy, diabetes risk	A to Z, PROVE-IT TIMI 22, IDEAL
Ezetimibe	NPC1L1 inhibition	Additive 20–24% LDL-C reduction	Modest efficacy alone	IMPROVE-IT
PCSK9 inhibitor monoclonal antibody	LDL-R upregulation	53% LDL-C reduction, plaque regression	Cost, injection site reactions	FOURIER, ODYSSEY-Outcomes
Bempedoic acid	ACL inhibition	Oral, no muscle toxicity	Gout, limited outcome data	CLEAR Outcomes
Icosapent ethyl	TG reduction	20% reduction in CVD	No LDL-C lowering	REDUCE-IT

CVD: cardiovascular disease; PCSK9: Proprotein Convertase Subtilisin/Kexin type 9; ACL: ATP citrate lyase; NPC1L1: Niemann Pick C1-like; HMG-CoA: 3-hydroxy-3-methylglutaryl-CoA; LDL-R: low-density lipoprotein receptor.

**Table 2:** Studies demonstrating early versus late intensive lipid-lowering therapy.

Trial/ Registry	Timing of Intervention	Population/ Setting	Lipid-Lowering Strategy	Main Outcomes/Findings	Early vs. Late Insights
LAI-REACT [47]	Early (immediate)	Statin-naive ACS patients	Triple therapy: rosuvastatin, ezetimibe, bempedoic acid (REB)	Rapid and sustained LDL-C reduction: 59.3%, 62.3%, 61.6%, and 59.7%, at weeks 1, 2, 4, and 6, respectively (*p* < 0.001). Target LDL-C < 50 mg/dL achieved in 61.3% in first week, 72.9% in second week, 69.2% in third week, and 65.1% in fourth week.	Triple REB therapy quickly and effectively lowers LDL-C after ACS, with significant reductions seen in one week and maintained for six weeks.
EVOPACS [34]	Early (within 24 h)	ACS patients	PCSK9 inhibitor (evolocumab + high-intensity statins	Target levels achieved in >95% patients until 8 weeks. Combination therapy was safe and effective.	Early combination therapy helped in achieving recommended LDL targets early and was safe.
SWEDE-HEART REGISTRY [50]	Early vs. late (Registry)	Swedish ACS/MI patients	Statins + ezetimibe <12 week: early combination therapy 13 to 16 week: late combination therapy Statins alone: no combination	Early ezetimibe initiation linked to reduced mortality.	Early combination therapy was associated with a lower risk of major adverse cardiovascular events (MACE—including death, MI, and stroke) and cardiovascular death compared to delayed or no ezetimibe.
PACMAN-MI [11]	Early	Post-MI, early post-event	PCSK9 inhibitor (alirocumab) + statin	Early PCSK9 inhibitor reduces plaque volume and improves stabilization in acute MI patients.	Early lowering improves plaque regression.
HUYGENS [12]	Early	Post-MI, early post-event	PCSK9 inhibitor (alirocumab) + statin	Early PCSK9 inhibitor improves plaque characteristics and reduces inflammation.	Early intervention shows favorable vascular effects.

## Data Availability

Not applicable.
